# Numerical analysis of single-point spectroscopy curves used in photo-carrier dynamics measurements by Kelvin probe force microscopy under frequency-modulated excitation

**DOI:** 10.3762/bjnano.9.175

**Published:** 2018-06-20

**Authors:** Pablo A Fernández Garrillo, Benjamin Grévin, Łukasz Borowik

**Affiliations:** 1 Univ. Grenoble Alpes, CEA, LETI, 38000 Grenoble, France; 2 Univ. Grenoble Alpes, CNRS, CEA, INAC, SYMNES, 38000 Grenoble, France

**Keywords:** carrier dynamics, carrier lifetime, carrier recombination, Kelvin probe force microscopy, nanostructured photovoltaics, numerical simulations, photo-carrier dynamics

## Abstract

In recent years, the investigation of the complex interplay between the nanostructure and photo-transport mechanisms has become of crucial importance for the development of many emerging photovoltaic technologies. In this context, Kelvin probe force microscopy under frequency-modulated excitation has emerged as a useful technique for probing photo-carrier dynamics and gaining access to carrier lifetime at the nanoscale in a wide range of photovoltaic materials. However, some aspects about the data interpretation of techniques based on this approach are still the subject of debate, for example, the plausible presence of capacitance artifacts. Special attention shall also be given to the mathematical model used in the data-fitting process as it constitutes a determining aspect in the calculation of time constants. Here, we propose and demonstrate an automatic numerical simulation routine that enables to predict the behavior of spectroscopy curves of the average surface photovoltage as a function of a frequency-modulated excitation source in photovoltaic materials, enabling to compare simulations and experimental results. We describe the general aspects of this simulation routine and we compare it against experimental results previously obtained using single-point Kelvin probe force microscopy under frequency-modulated excitation over a silicon nanocrystal solar cell, as well as against results obtained by intensity-modulated scanning Kelvin probe microscopy over a polymer/fullerene bulk heterojunction device. Moreover, we show how this simulation routine can complement experimental results as additional information about the photo-carrier dynamics of the sample can be gained via the numerical analysis.

## Introduction

In the past decade, the nanoscale investigation of materials properties has captured the attention of the scientific community, partially due to its crucial importance in the improvement of photovoltaic devices [1–2]. Carrier lifetime, or more broadly speaking, photo-carrier dynamics is one of the most interesting parameters to study at the local scale. To date, various questions regarding the interplay between photo-carrier dynamics and structuration of materials remain unanswered, and it is not clear how it affects performances in some emerging photovoltaic technologies.

In this context, few teams around the world have recently began to develop time-resolved scanning probe microscopies (SPM) techniques, aimed at addressing the photo-carrier dynamics at the local scale in photoactive materials and devices. At this point, Kelvin probe force microscopy (KPFM) emerged as a useful technique that, when implemented under frequency-modulated excitation, can be used to investigate the surface photovoltage decay, thus providing access to the photo-carrier dynamics [3–11].

A common aspect among all KPFM frequency-modulated spectroscopy techniques is that in order to extract time constants associated to photo**-**physical processes, a mathematical fit procedure is usually implemented. It is evident that the mathematical model used in the fit procedure constitutes a determining aspect in the calculation of time constants. Hence, there is a need to define methods that could check the validity of the mathematical assumptions. This led us to develop a simulation routine that enables to predict the behavior of spectroscopy curves of the average photovoltage as a function of a frequency-modulated excitation source in photovoltaic materials.

In this paper, we describe the general aspects of this simulation routine, and we compare it against experimental results from a previous work were single-point Kelvin probe force microscopy under frequency-modulated illumination (FMI-KPFM) was implemented over a silicon nanocrystal solar cell [3]. Analogously, we compare the simulation routine against the results obtained by intensity-modulated scanning Kelvin probe microscopy on a polymer/fullerene bulk heterojunction device as presented by Shao and co-workers [4]. The outcome of these comparisons did not only provide additional evidence supporting results obtained using the abovementioned techniques as simulations displayed a good agreement with experimental measurements. It also revealed that the simulation routine can complement experimental results as additional information about the photo-carrier dynamics of the sample can be gained through numerical analysis.

## Experimental

Photo-carrier generation is a process that takes place in semiconductor materials when electron–hole pairs (positive and negative polarons in the case of organic photovoltaics) are created by exciting an electron from the valence band to the conduction band (π-electrons from the highest occupied molecular orbital of the molecule to the lowest unoccupied molecular orbital in the case of organic photovoltaics), thus leaving a hole behind that can be considered as a positive charge. Recombination is the opposed process where negative and positive charges recombine and are annihilated.

In both cases, when the system is supplied with additional energy, i.e., through photon absorption, additional carriers are generated. In photovoltaic devices, an open-circuit voltage (*V*_OC_) appears when carriers are photo-generated. In the same way, carrier recombination occurs when the extra energy is no longer supplied to the system and *V*_OC_ decays until the charge equilibrium state is reached.

**Figure 1:**
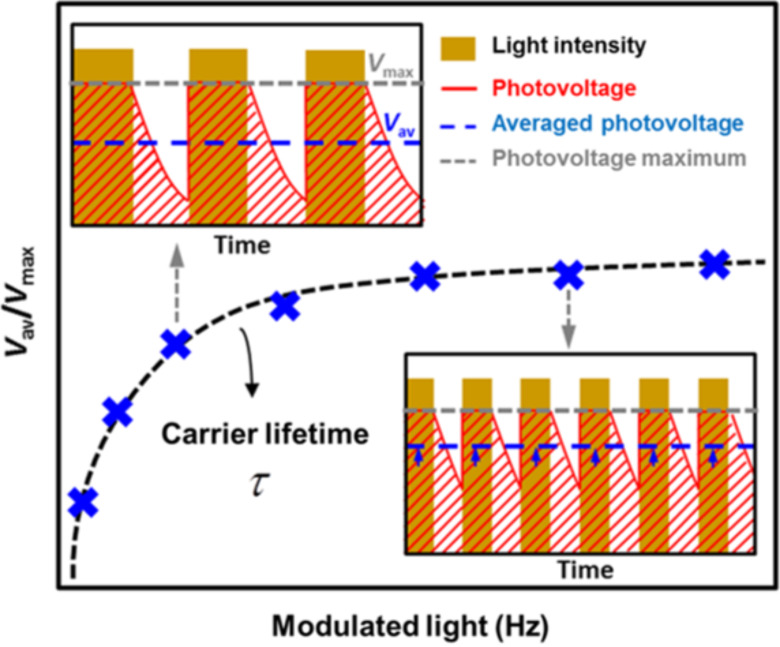
FMI-KPFM working principle: Averaged time-integral values of the instantaneous photovoltage are acquired as a function of the excitation-modulation frequency. SPV decay time constants are calculated upon a mathematical fit of this curve. In FMI-KPFM, images of the SPV decay time constant can be acquired by simultaneously performing this protocol over multiples points on a pre-defined grid area over the sample [3–6].

The surface photovoltage (SPV), which can be seen as a local measurement of *V*_OC_ in semiconductors [12], has been studied using KPFM under modulated illumination. Indeed, the investigation of the SPV evolution as a function of a frequency-modulated excitation source can be used to access the photo-carrier dynamics in organic, inorganic and hybrid semiconductors [3–9,13]. In short, as depicted in Figure 1, FMI-KPFM consist of the measurement of a surface photovoltage by KPFM (time response between a few milliseconds and a few hundreds of milleseconds) under frequency-modulated excitation (light source, electrical bias), yielding an averaged time-integral value of the instantaneous photovoltage. One can obtain a spectroscopy curve of the average surface photovoltage (SPV_AV_) by sweeping the excitation source modulation frequency. The spectroscopy curve is then fitted using mathematical models that enable one to determine the time constant(s) associated to the measured SPV dynamics. One of the advantages of FMI-KPFM compared to similar techniques is that in FMI-KPFM, images of the SPV decay time constant can be acquired by simultaneously performing the above describe protocol over multiples points on a pre-defined grid area over the sample. However, in the following, the discussion of FMI-KPFM results will turn around single-point measurements.

For the most part, techniques based on this approach do not take into account the built-up time of the SPV, which is the time needed for the surface photovoltage to appear in the first place. This time is associated with the exciton generation, charge dissociation and charge transport along the material so that a photo-generated surface potential can be detected using KPFM. Indeed, in some cases, depending on the material and the excitation intensity, this time can be approximated to zero as the SPV built-up is usually much faster than the SPV decay. However, we will see below that taking into account a non-zero SPV built-up time can modify the interpretation of the spectroscopy curves and, thus, the estimation of the SPV time constants. Here we highlight that the SPV built-up time can be physically interpreted in different ways depending on the sample and the photo-generation mechanism. In inorganic silicon samples for instance, this time constant can be attributed to the effective time needed for exciton generation, dissociation and carrier separation. On the other hand, in the case of some organic photovoltaic (OPV) samples, the SPV built-up time can be attributed to the effective time needed to fill lower energy states (traps). In a more general way, it can be stated that the SPV built-up time is closely related to the carrier diffusion length within each particular material.

In a previous work [1], we implemented a single exponential decay model to fit the spectroscopy curves acquired over a silicon nanocrystal solar cell. In the following, using a novel automated numerical analysis routine, we verify the validity of the model by checking the self-consistency of the previously obtained results via the comparison of measured data, mathematical fit and simulations.

In a first approach, an exponential function can be used to describe the built-up and decay of the SPV in photoactive materials [3–6,8]. Under this premise, we can model the SPV behavior of a photovoltaic material under modulated excitation as a function of the time for both the built-up and decay in the following way for the case of a single SPV built-up and decay time constant (Equation 1 and Equation 2) and for a more general case with *k* build-up and *l* decay time constants (Equation 3 and Equation 4):

[1]
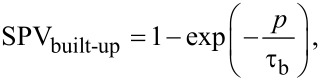


[2]
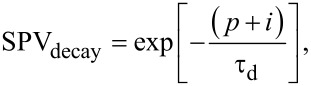


[3]
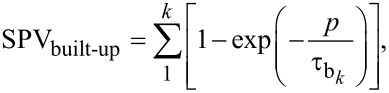


[4]
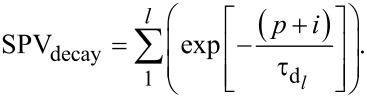


Here, *p* is the time duration of the excitation pulse, *i* is the duration of time between the pulses, τ_b_ is the time constant associated to SPV built-up, and τ_d_ is the time constant associated to SPV decay.

Here, the number of build-up and decay time constants can be determined in advance from accessible literature by taking to account the type and properties of materials, e.g., doping level and type, defects concentration and gap energy. Types of recombination mechanisms for silicon can be found in [14].

**Figure 2:**
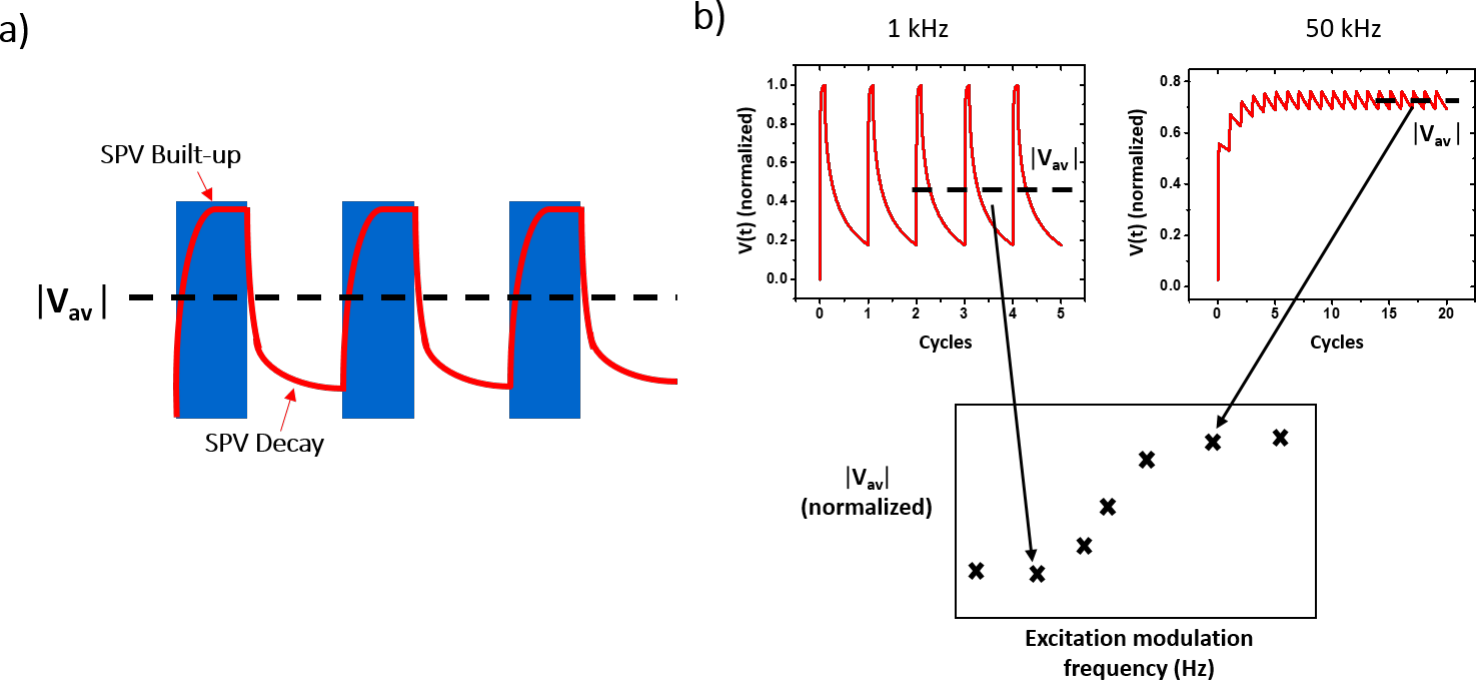
SPECTY working principle: (a) SPV built-up and decay are modeled using Equation 1 and Equation 2 respectively. (b) A quasi-steady-state condition is reached after a certain number of excitation pulses. At this moment the average value of the normalized SPV is calculated. Performing this calculation at different modulation frequencies allows one to plot the evolution of the normalized average surface photovoltage magnitude as a function of the excitation modulation frequency.

Using Equation 1 and Equation 2 we can numerically model the normalized magnitude of the instant SPV as a function of the time for different excitation modulation conditions (Figure 2a). In other words, we can access the normalized magnitude of the instant SPV at any given point in time for any given modulation frequency. Depending on the imposed modulation frequency value, a quasi-steady-state condition is reached after a certain number of excitation pulses, indicating that the equilibrium state of charges was reached. Once this condition is attained, we calculate the average value of the normalized SPV through integration. By performing this calculation at different modulation frequencies we can then plot the evolution of the normalized average surface photovoltage magnitude as a function of the excitation modulation frequency as depicted in Figure 2b. A custom-written software (SPECTY) implementing this routine was separately developed using the SCILAB open source coding tool and the batch processing options of OriginPro software (OriginLab Corp.) yielding the same results.

**Figure 3:**
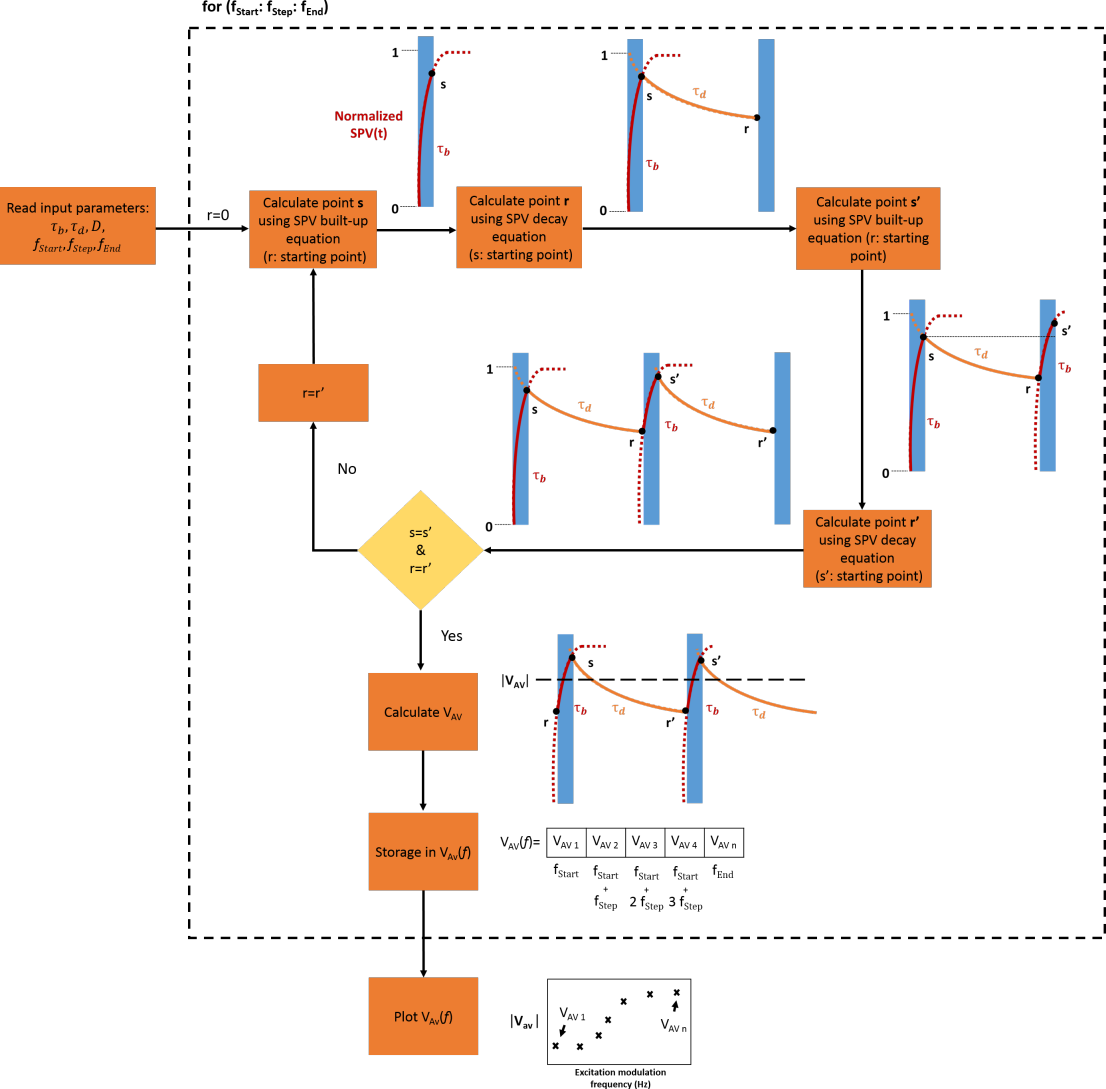
SPECTY’s algorithm flowchart. When a quasi-steady-state condition is reached, the average value of the normalized surface potential (*V*_AV_) is calculated.

While both the syntax and the philosophy of the SCILAB open source coding tool and the batch processing options of OriginPro software are different, in both coding environments SPECTY is structured in a similar way. Figure 3 depicts the software flowchart detailing the algorithm used in the performed simulations. As depicted in this figure, upon the introduction of the simulation input parameters (SPV decay and built-up time constants along with the duty ratio and the range of frequencies), the software applies Equation 1 to find the attained value of the surface photovoltage just at the end of the illumination period (called “s” in Figure 3), then in a similar way, the software uses Equation 2 to find the attained value of the surface photovoltage after photo-carrier recombination during the “in-dark” period (called “r” in Figure 3). This process is repeated until two consecutive “s” and “r” points have the same value, which means that the quasi-steady-state condition was reached. Then, the average value of the last two pulses is calculated and stored in the form of a vector. At the end of the for loop, this vector is plotted yielding the spectroscopy curve *V*_AV_(*f*).

## Results and Discussion

Using SPECTY we can now verify the validity of the mathematical model used in a previous work, where the minority-carrier lifetime in a silicon nanocrystal solar cell was obtained by KPFM spectroscopy under frequency-modulated light illumination [3]. This can be done by fixing the SPV decay time in the numerical simulation to the value predicted by the mathematical fit used on that occasion and comparing the correspondence between the spectroscopy curve resulting from the mathematical fit and the data points obtained from the numerical simulation.

In [3], minority-carrier lifetime values were calculated through a mathematical fit procedure derived from previous publications [5] using the following expression:

[5]
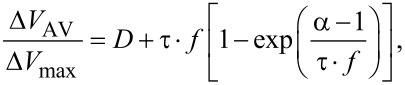


where *D* is the modulation duty ratio, *f* is the modulation frequency of the light, (Δ*V*_AV_/Δ*V*_max_) is the ratio between the time-averaged surface potential and the surface photovoltage at saturation, and τ is the minority-carrier lifetime.

**Figure 4:**
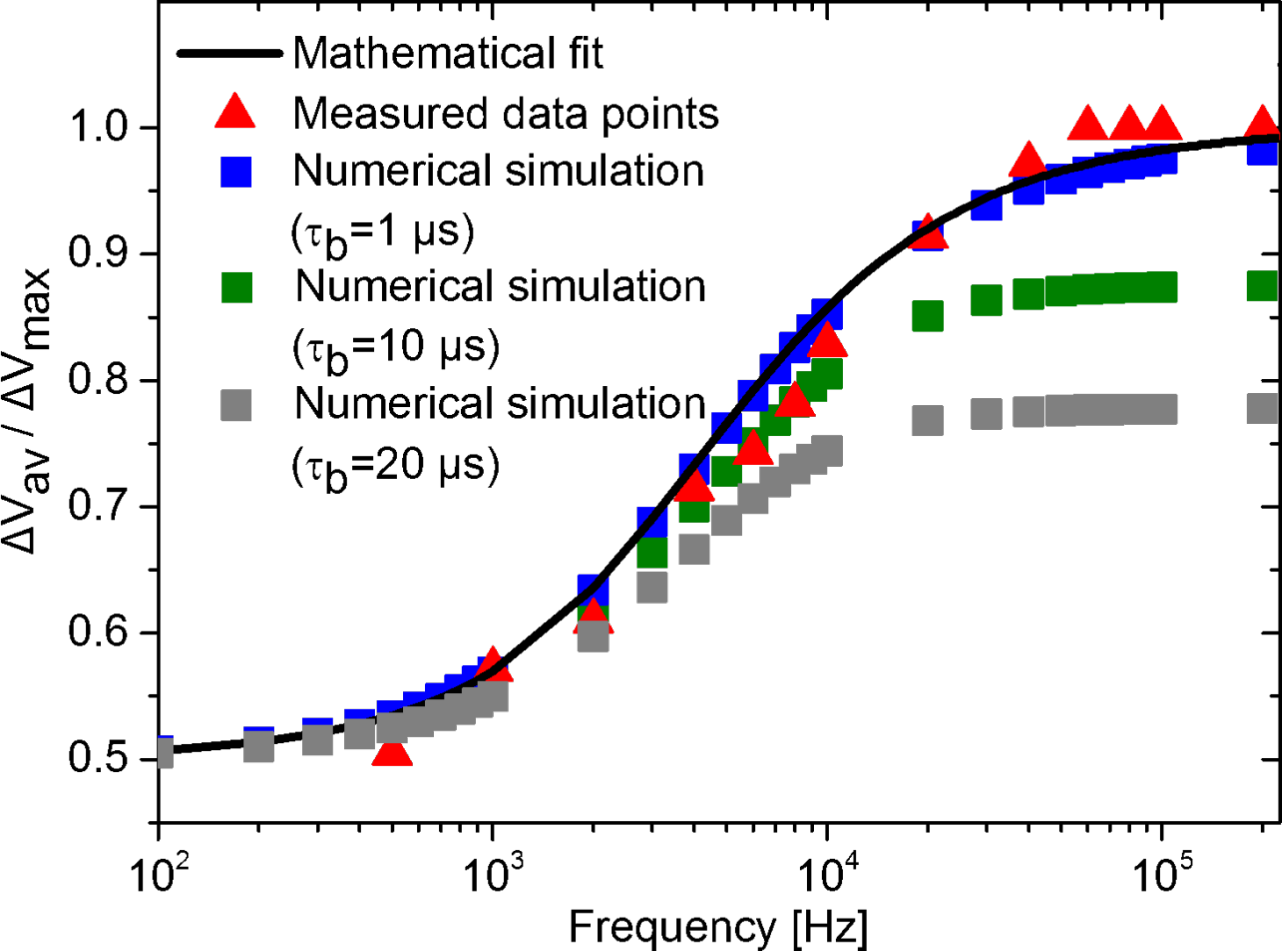
Comparison between spectroscopy curve obtained through the mathematical fit, measured data points and data points obtained by numerical simulations using τ_b_ = 1, 10 and 20 μs. The data points was taken from Figure 4 of [3].

Figure 4 shows the spectroscopy curve resulting from the mathematical fit from which the minority-carrier lifetime was extracted in a silicon nanocrystal solar cell after H-passivation along with the measured data points as presented in [3] together with the simulated data points. For the numerical simulation, τ_d_ was fixed at 70 µs (to match the value predicted by the mathematical fit), τ_b_ was fixed at 1 µs, but similar results were obtained using shorter values. On the other hand, the use of τ_b_ > 1 μs yields simulated data points that no longer follow the mathematical fit curve (green and gray squares in Figure 4). Both *p* and *i* were chosen to match the experimental parameters used in [3].

In Figure 4 we observe a full correspondence between the mathematical fit applied to the minority-carrier lifetime in the silicon nanocrystal solar cell after H-passivation and the numerically simulated data points (τ_b_ = 1 μs). In addition, this routine provides additional information about the SPV built-up time constant, as it shall be 1 µs at most so that the simulation agrees with the measured data points as shown in Figure 4. This information would not otherwise be accessible solely from the mathematical fit used in our previous paper [3]. Moreover, this time constant value strongly agrees with previous reports of the time scale of photo-generation and electron–hole pair separation in other silicon samples [9,15–16].

**Figure 5:**
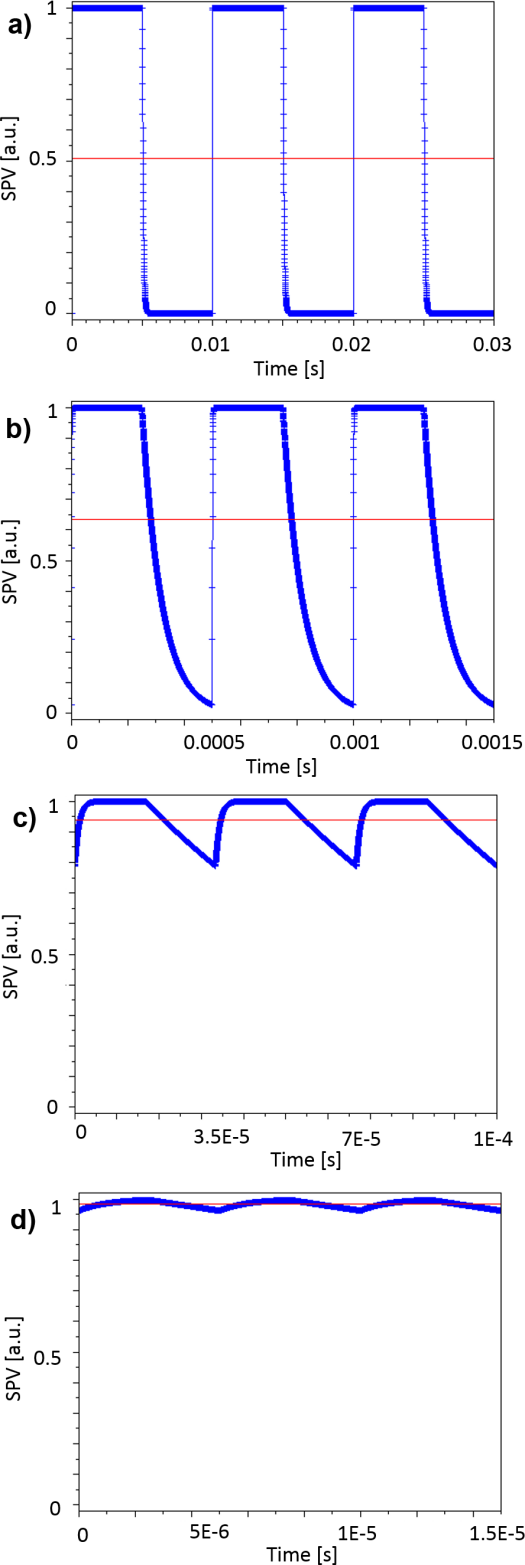
Simulated SPV as a function of the time for different excitation modulation frequencies, the red lines indicate the average SPV value for each modulation condition. Modulation frequencies: a) 100 Hz, b) 2 kHz, c) 30 kHz, and d) 200 kHz.

Additionally, SPECTY can provide graphic representations of how the SPV as a function of the time evolves with the modulation frequency as shown in Figure 5.

**Figure 6:**
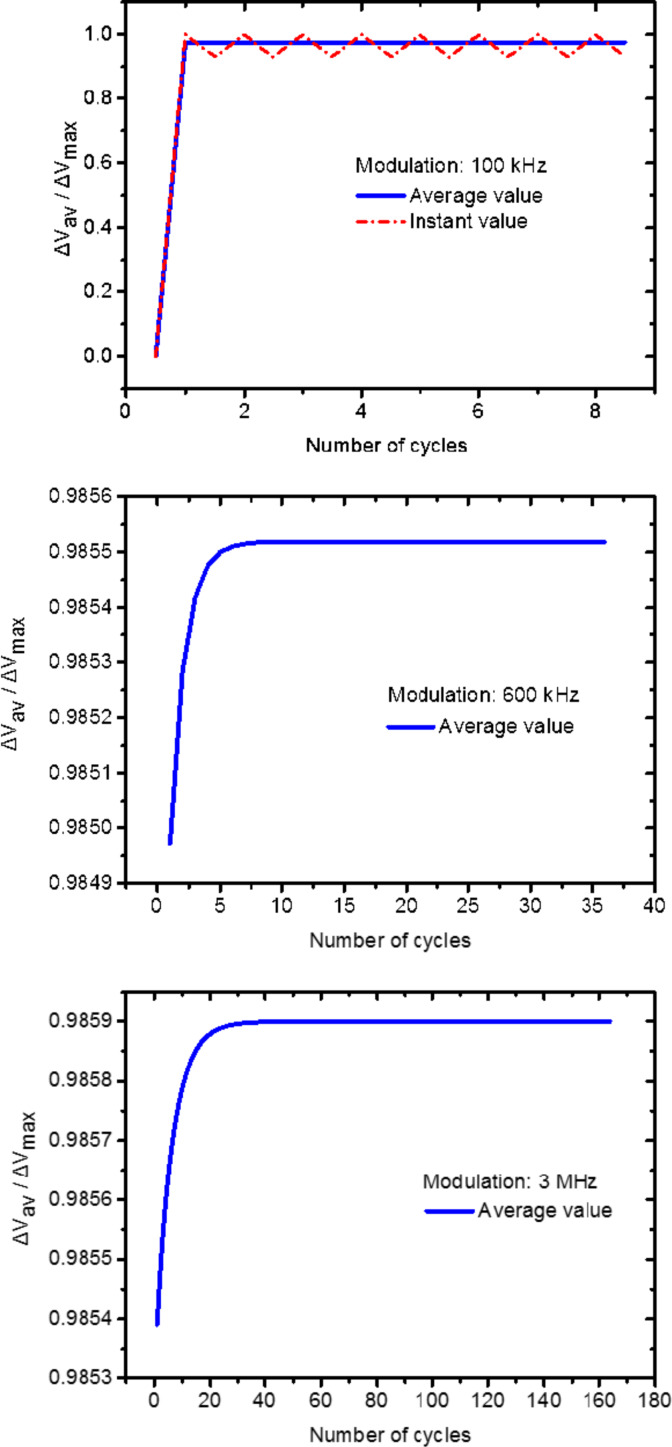
Simulated SPV_AV_ as a function of the number of excitation cycles for different excitation modulation frequencies. From top to bottom: 100 kHz, 600 kHz and 3 MHz. The simulation at 100 kHz includes the SPV instant value oscillation as depicted by the red dashed line. The graphs depicting the results from simulations at 600 kHz and 3 MHz were zoomed-in so that the convergence process can be noticed.

Information about the evolution of SPV_AV_ as a function of the number of excitation cycles can also be accessed via the simulation routine as depicted in Figure 6. In fact, as described above, depending on the value of the imposed modulation frequency, a quasi-steady-state condition is reached after a certain number of excitation pulses (charge equilibrium). In Figure 6 we note that as expected, the higher the modulation frequency is the more illumination cycles are needed to attain the charge-equilibrium state. Nonetheless, we stress that even though more cycles are needed to attain this condition, in terms of time it remains negligible compared to the KPFM integration time.

After having demonstrated how to apply the numerical analysis routine in single-point FMI-KPFM results obtained over a silicon nanocrystal solar cell, we now turn to the analysis of results obtained by intensity-modulated scanning Kelvin probe microscopy over a polymer/fullerene bulk heterojunction device as presented by Shao and co-workers [4].

As stated above, SPECTY can be useful in the analysis of results obtained by several frequency-modulated KPFM techniques. Intensity-modulated scanning Kelvin probe microscopy is a technique that allows one to study the surface photovoltage decay on sub-millisecond time scales in photovoltaic materials. This technique [4], was used to measure the local photo-carrier lifetime over a region of a PCDTBT/PC_71_BM bulk heterojunction sample that had either 2,6-difluorobenzylphosphonic acid (oF_2_BnPA) or pentafluorobenzylphosphonic acid (F_5_BnPA) underneath. In this work it was found that the characteristic photo-carrier lifetime was about two times faster for oF_2_BnPA than for F_5_BnPA regions at a given light intensity as the characteristic photo-carrier lifetime values extracted from the raw data were 0.51 ms and 1.1 ms, respectively. In the work of Shao and co-workers, a stretched exponential function was used in the fit procedure to describe the dispersive kinetics nature of the SPV decay where the lifetime changes with time.

In the following, using same data, as extracted from Figure 8 of [4], we propose instead, the use of exponential functions including a non-zero SPV built-up time to simulate the resulting average surface photovoltage spectroscopy curves for F_5_BnPA and oF_2_BnPA regions.

**Figure 7:**
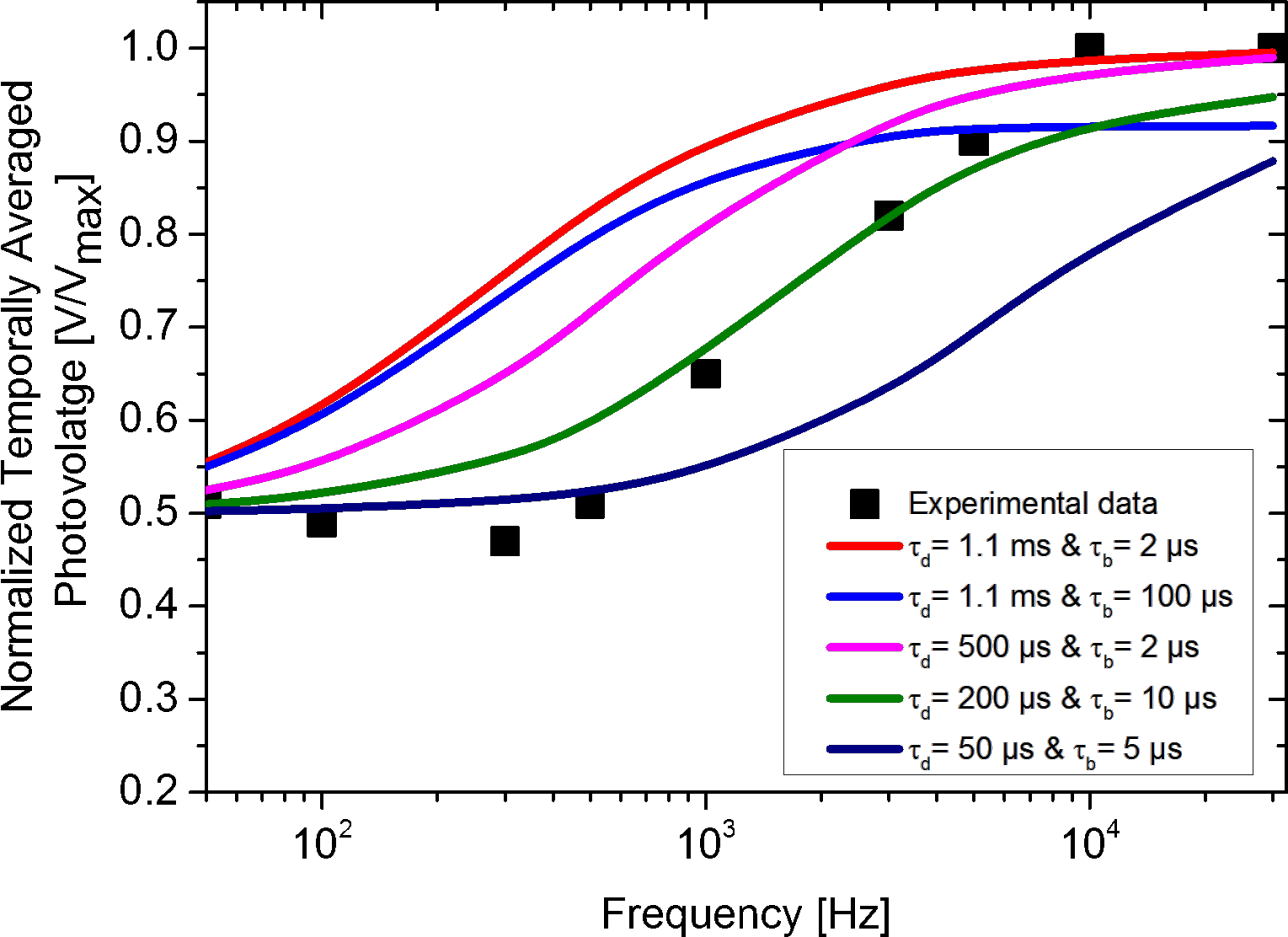
Simulated surface photovoltage spectroscopy curves of the F_5_BnPA region for different SPV built-up and decay input parameters. Experimental data was extracted from Figure 8 of [4].

Figure 7 shows different simulated surface photovoltage spectroscopy curves for the F5BnPA region. Based on the analysis of this figure, it can be suggested that the inclusion of a non-zero SPV built-up time demands the use of a shorter SPV decay time as input parameter to the simulation (compared to the 1.1 ms found using a stretched exponential function for fitting purposes), as well as a SPV built-up time ≤2 µs in order to simulate a surface photovoltage spectroscopy curve that passes through the data points with minimum deviation. Similar results were obtained for the oF_2_BnPA region (not shown).

**Figure 8:**
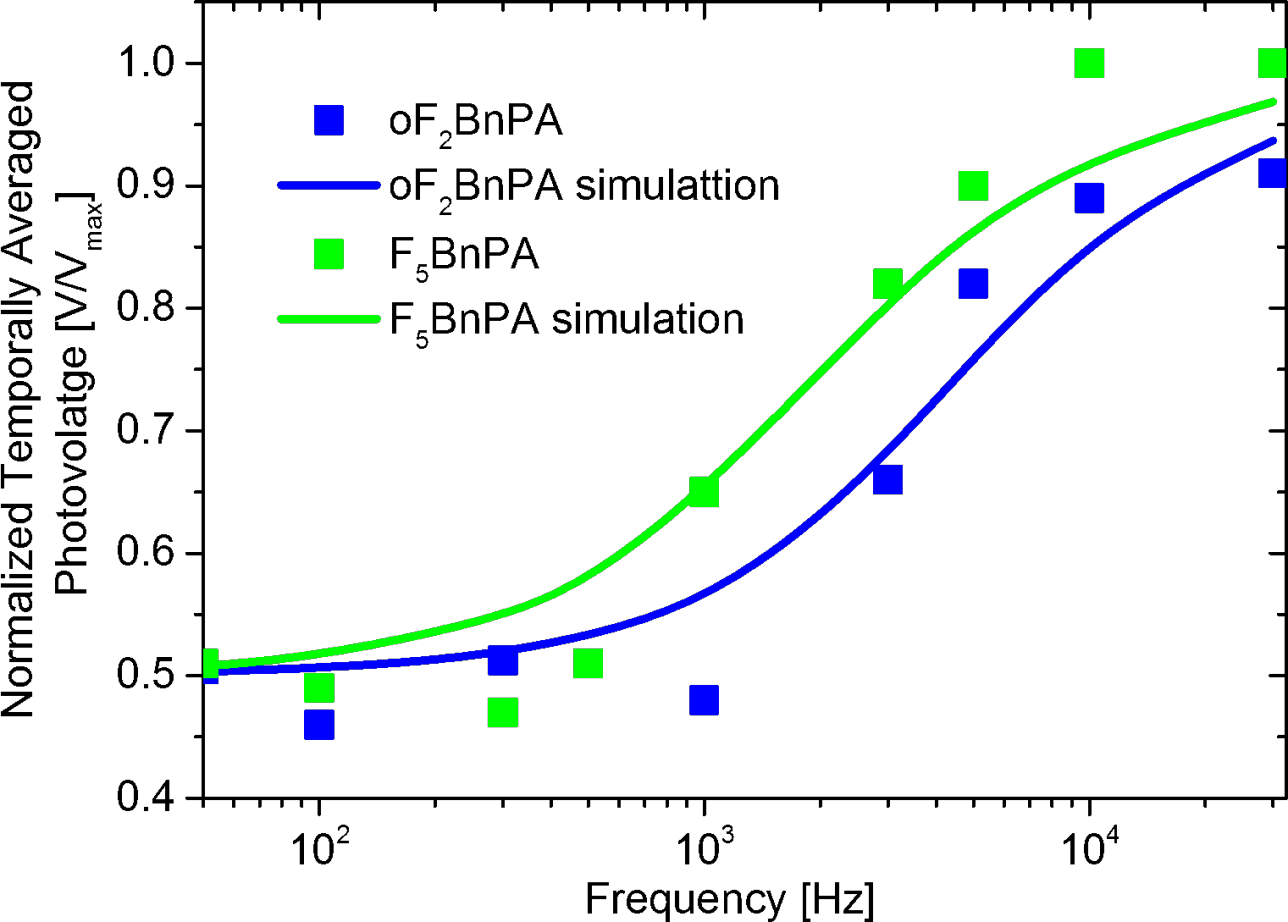
Time-averaged surface photovoltage measured at different modulating frequencies with intensity-modulated scanning Kelvin probe microscopy. Blue and green dots are experimental data for the oF_2_BnPA and F_5_BnPA areas, respectively. Blue and green lines are best obtained results of the simulated average surface photovoltage spectroscopy curves obtained with SPECTY using τ_d_ = 68.1 μs and τ_b_ = 1 μs for the oF_2_BnPA region, and τ_d_ = 158.2 μs and τ_b_ = 2 μs for the F_5_BnPA region as input parameters. Experimental data points were taken from Figure 8 of [4].

Figure 8 displays the best obtained result of the simulated average surface photovoltage spectroscopy curves and the measured data points for both F_5_BnPA and oF_2_BnPA regions. The spectroscopy curves presented in Figure 8 were obtained with SPECTY using τ_d_ = 68.1 μs and τ_b_ = 1 μs for the oF_2_BnPA region, and τ_d_ = 158.2 μs and τ_b_ = 2 μs for the F_5_BnPA region as input parameters, in both cases *p* and *i* were chosen to match the experimental parameters used in [4].

While the simulated results presented in Figure 8 resemble those from [4], we nonetheless note that the adjustment level of the simulated curves to the data points does not allow to conclusively claim that estimated SPV time constants are indeed representing the photo-carrier dynamics of the sample. Indeed, it would appear that simulated curves tend to increase even further for higher frequencies while the experimental points reach a plateau, this suggest that more complex photo-carrier dynamics are governing the SPV behavior of the sample, as it is the case for instance for OPV samples exhibiting a high density of low-energy states (traps).

Nonetheless, we stress that even if the calculated photo-carrier lifetime values do not fully agree with those reported in [4], the ratio between the calculated photo-carrier lifetime in F_5_BnPA and oF_2_BnPA regions remains the same (the characteristic carrier lifetimes of oF_2_BnPA are about half than those of F_5_BnPA).

Moreover, even if the results in Figure 8 are not entirely conclusive, the measured time scales for the SPV dynamics do seem to agree relatively well with the results of macroscopic transient experiments over similar samples [17]. However, the discrepancy between these results in the microsecond range and those reported previously in the millisecond range, opens a debate around whether the measured intensity-modulated scanning Kelvin probe microscopy data should be analyzed using stretched exponentials with a certain stretching exponent to describe the dispersive kinetics where the lifetime changes with time, or rather use an exponential function that accounts for a non-zero surface photovoltage built-up time.

While there is no short answer to this question, here we highlight that when using stretched exponentials in order to take into account the dispersive kinetics present in the sample, the stretch exponents lie between 0 and 1 [18–21]. However, in [4] the stretching exponent that best fits the results is greater than 1. This inconsistency put some constrains on the interpretation of the calculated photo-carrier time constants.

In order to determine which mathematical model describes best the physical phenomena occurring in the sample upon photo-carrier generation, an experimental protocol is proposed hereafter. Measuring the contact potential difference (CPD) under continuous wave illumination (or DC bias excitation) can give us the magnitude of the average potential that we should detect for the highest modulation frequency if the SPV built-up time can be approximated to zero. In this scenario, data can be fitted assuming τ_b_ = 0. On the other hand, if the average potential measured for the highest modulation frequency, is below the CPD under continuous wave excitation previously registered, a non-zero SPV built-up time needs to be assumed.

To demonstrate this, a new FMI-KPFM acquisition protocol was developed, in which both the CPD under continuous wave excitation and the SPV_AV_ spectroscopy curve can be simultaneously acquired at each point of the sample. Indeed, by applying a continuous wave excitation pulse to the sample, prior to the acquisition of the SPV_AV_ spectroscopy curve, it becomes possible to measure values of both the CPD in dark conditions and under continuous wave illumination.

**Figure 9:**
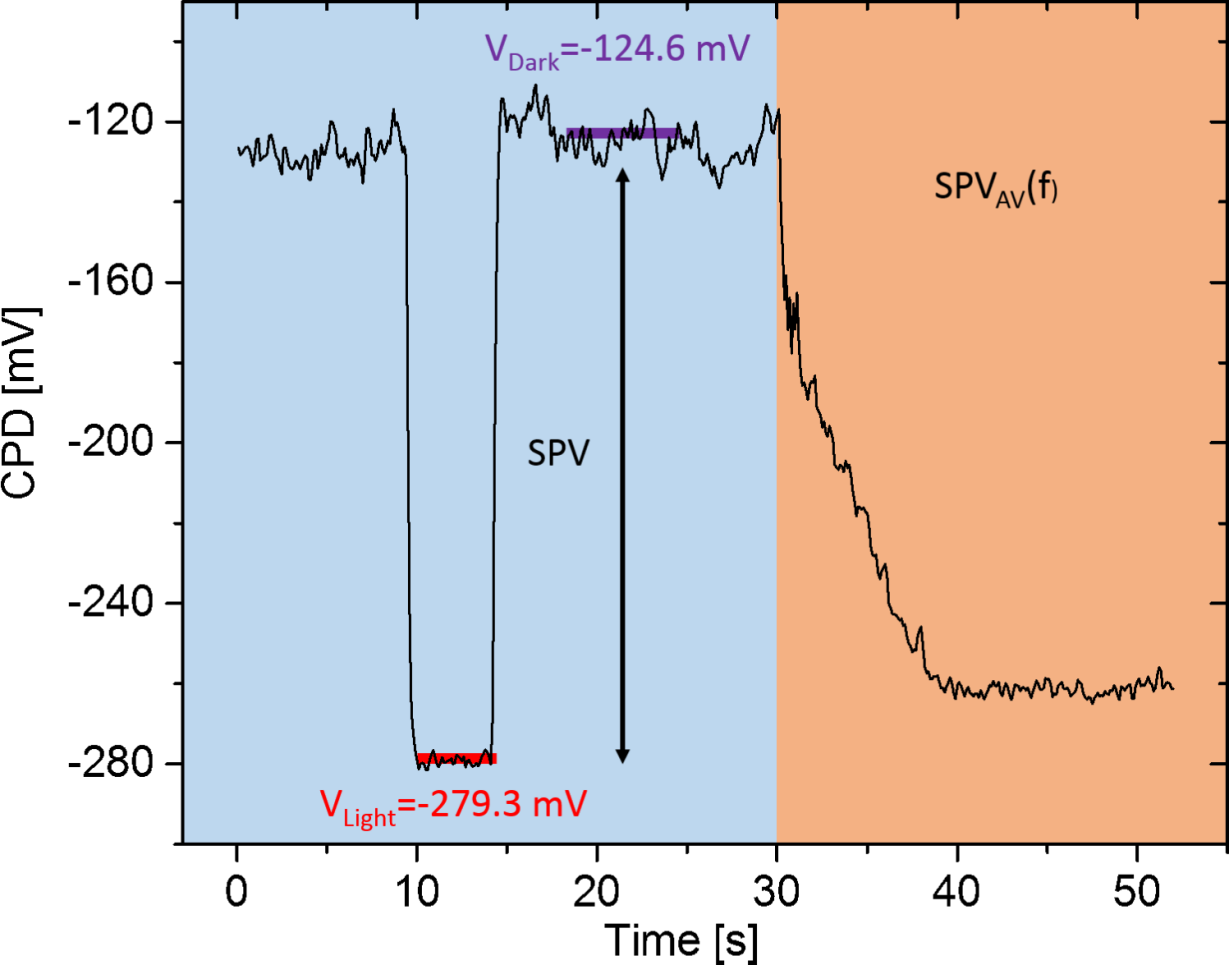
Data obtained from the implementation of the proposed FMI-KPFM protocol for the simultaneous acquisition of the CPD under continuous wave excitation and under dark conditions, along with the SPV_AV_ spectroscopy curve. *V*_Dark_ corresponds to the in-dark surface potential and *V*_Light_ is the surface photovoltage measured under continuous wave illumination.

Figure 9 shows an example of the obtained result when implementing this protocol over a nano-phase segregated PDBSTQx/PC_71_BM blend with amplitude modulation FMI-KPFM. In this case, the sample was optically excited using a green (515 nm) PhoxXplus module from OmicronLaserage GmbH (rise and fall times <1.5 ns in digital modulation mode) with a peak output power of 50 mW/cm^2^. Modulation frequencies were swept from few tens of hertz to 10 kHz with a 10% duty ratio. We highlight that this sample was previously investigated by our group in an earlier work [6]. However, its photo-physical properties evolved after near 20 months of storage time under UHV conditions.

**Figure 10:**
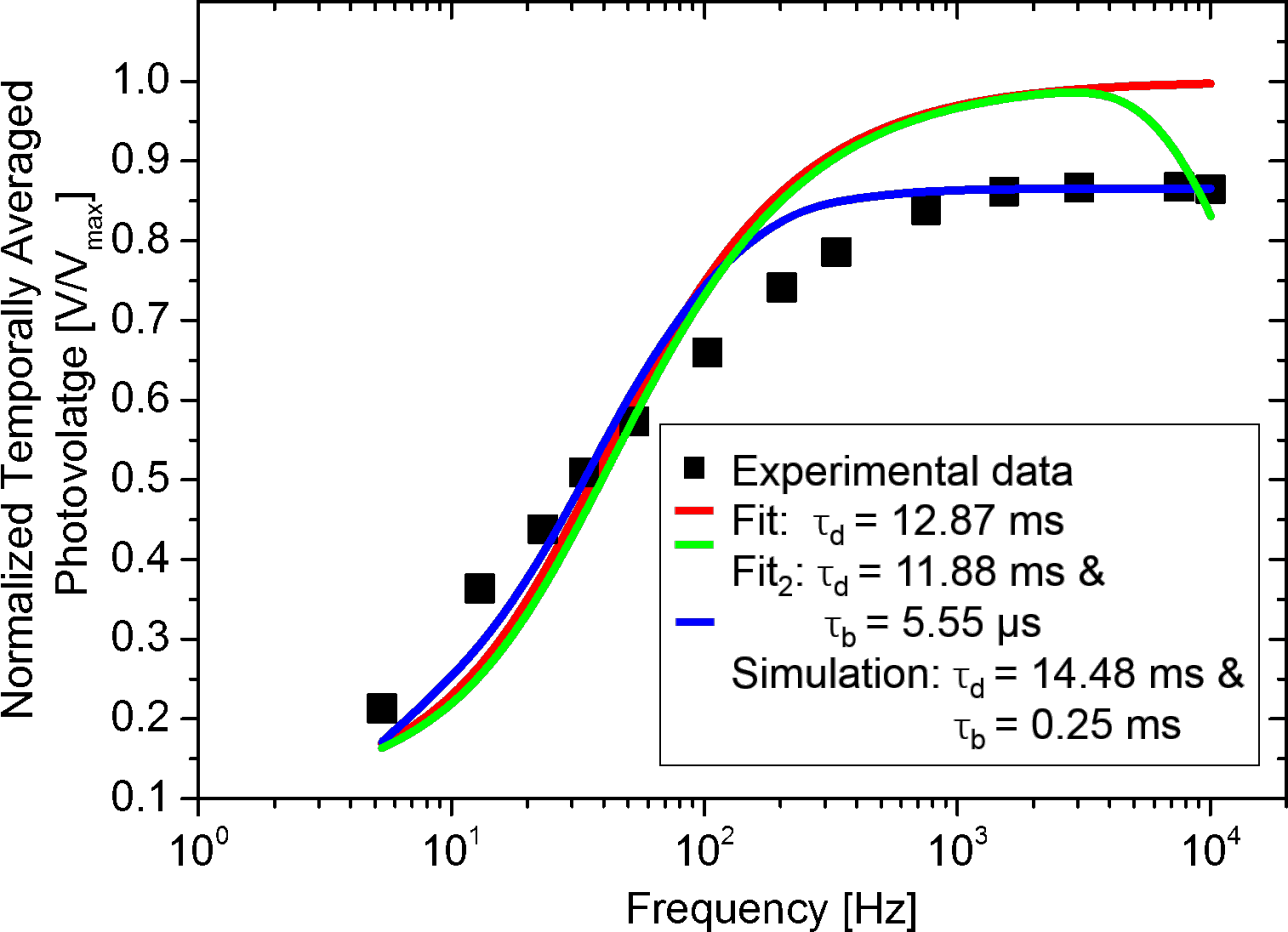
Comparison between curves: Mathematical fit (red and green lines) and simulated SPV_AV_ curve (blue line). The improved adjustment of the simulated SPV_AV_ curve compared to the mathematical fit is attributed to the inclusion of a non-zero SPV built-up time.

As it can be seen from Figure 9, the magnitude of the CPD measured under continuous wave illumination is higher than the average potential measured at the highest modulation frequency. As mentioned before, in this scenario we propose that a non-zero SPV built-up time shall be accounted for. To do so, firstly the normalized SPV_AV_ spectroscopy curve is extracted as shown in Figure 10. As a first step, we fitted this SPV_AV_ spectroscopy curve using the following equations derived from a previous work to estimate separately the SPV decay time constant τ_d_ and also τ_d_ together with a non-zero SPV built-up time constant τ_b_ [4]:

[6]
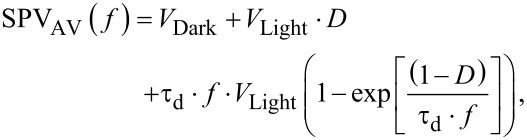


[7]
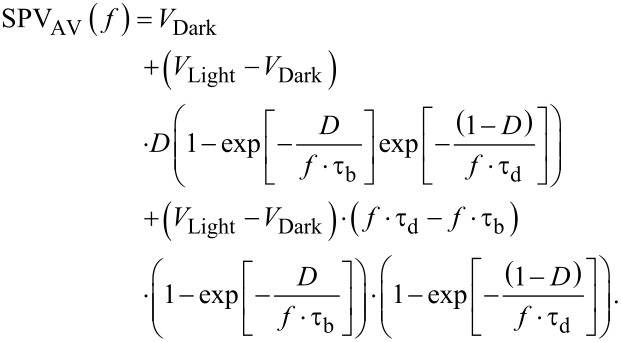


In these expressions *V*_Dark_ is the in-dark surface potential, *V*_Light_ the surface photovoltage measured under continuous wave illumination, τ_d_ the SPV decay time, *f* is the modulation frequency and *D* is the illumination duty ratio. Note that Equation 6 does not take into account a non-zero SPV built-up time, in contrast to Equation 7. In both expressions *V*_Dark_ and *V*_Light_ are known values that can be set constant in the fit procedure.

In Figure 10, the results of the fit procedures are shown (red and green lines). Although a SPV decay time within the expected range is calculated (ca. 12 ms), the fits exhibit large deviations from the data points. In turn, if we use SPECTY to model the SPV_AV_ spectroscopy curve including the use of a non-zero SPV built-up time, the resulting curve (blue line) presents an improved adjustment to the data points, suggesting that indeed, using SPECTY leads to a more accurate estimation of the SPV photo-carrier dynamics.

As in Figure 8, the blue line in Figure 10 displays the best obtained result of the simulated average surface photovoltage spectroscopy curve over the measured data points using using τ_d_ = 14.48 ms and τ_b_ = 0.25 ms. However, it is worth mentioning that while the simulated curve seems to better describe the data set than the mathematical fit, all estimations yield similar SPV decay times.

## Conclusion

In summary, we proposed and demonstrated a novel automatic numerical simulation routine that enables the simulation of spectroscopy curves of the average surface photovoltage during the frequency-modulated excitation of photovoltaic materials, provided that the values of the time constants of the SPV dynamics are specified as set-up parameters in the software.

We implemented this routine to check calculated time constants associated to the minority-carrier lifetime obtained with single-point FMI-KPFM on a silicon nanocrystal solar cell. The obtained results were not only confirmed by the numerical analysis, but additional information about the photo-carrier dynamics was found, which otherwise would not be accessible solely from the mathematical fit of the measured data.

We also implemented a numerical simulation routine to check the pertinence of the mathematical model used in intensity-modulated scanning Kelvin probe microscopy measurements of a polymer/fullerene bulk heterojunction device. The output of this analysis led us to propose and demonstrate an experimental protocol for FMI-KPFM and related techniques, intended to help choosing the most adequate mathematical model for a given data set based on the nature of the SPV built-up time.
